# *Avena fatua* caryopsis dormancy release is associated with changes in KAR_1_ and ABA sensitivity as well as with ABA reduction in coleorhiza and radicle

**DOI:** 10.1007/s00425-020-03562-4

**Published:** 2021-01-28

**Authors:** Jan Kępczyński, Agata Wójcik, Michał Dziurka

**Affiliations:** 1 Institute of Biology, University of Szczecin, Wąska 13, 71-415 Szczecin, Poland; 2 Institute of Plant Physiology, Polish Academy of Sciences, Niezapominajek 21, 20-239 Krakow, Poland

**Keywords:** Abscisic acid, After-ripening, *Avena fatua*, Cell cycle, Coleorhiza, Dormancy, Karrikin, Radicle

## Abstract

**Main conclusion:**

The dormancy release in *Avena fatua c*aryopses was associated with a reduction in the ABA content in embryos, coleorhiza and radicle. The coleorhiza proved more sensitive to KAR_1_ and less sensitive to ABA than the radicle. The inability of dormant caryopses and ABA-treated non-dormant caryopses to complete germination is related to inhibition and delayed of cell-cycle activation, respectively.

**Abstract:**

As freshly harvested *Avena fatua* caryopses are dormant at 20 °C, they cannot complete germination; the radicle is not able to emerge. Both karrikin 1 (KAR_1_) and dry after-ripening release dormancy, enabling the emergence of, first, the coleorhiza and later the radicle. The after-ripening removes caryopse sensitivity to KAR_1_ and decreases the sensitivity to abscisic acid (ABA). The coleorhiza was found to be more sensitive to KAR_1_, and less sensitive to ABA, than radicles. Effects of KAR_1_ and after-ripening were associated with a reduction of the embryo’s ABA content during caryopsis germination. KAR_1_ was found to decrease the ABA content in the coleorhiza and radicles. Germination of after-ripened caryopses was associated with the progress of cell-cycle activation before coleorhiza emergence. Inhibition of the germination completion due to dormancy or treating the non-dormant caryopses with ABA was associated with a total and partial inhibition of cell-cycle activation, respectively.

## Introduction

Intact viable seeds of many plant species cannot complete germination after harvest, even under favourable conditions (water, temperature, air, and light). Such seeds are considered primarily dormant (Bewley 1997). Dormancy is absolute, when a seed cannot complete germination at all under any circumstances, or relative, if germination completion is possible only within a narrow range of conditions (Rodríguez et al. 2015). For example, caryopsis dormancy in temperate cereals is expressed at temperatures usually exceeding 15–20 °C, tropical cereals showing dormancy at temperatures lower than 25–30 °C (Simpson 2007; Rodríguez et al. 2015). Most dormant and non-dormant seeds can enter phase I, associated with a rapid water uptake, and phase II at which the water content is constant or increases slightly (Bewley et al. 2013). Phase III, associated with a further water uptake, post-germination processes, and seedling production, occurs in non-dormant seeds only. Germination of non-dormant seeds has been completed when the radicle or other embryonic tissue emerges through the structure covering it (Bewley et al. 2013). In grasses such as barley and *Brachypodium distachyon*, caryopsis germination can be regarded as a process consisting of two stages (González-Calle et al. 2015): the first associated with the appearance of the coleorhiza structure protecting the emerging radicle, and the second involving radicle emergence. Thus, just the emergence of the radicle through the coleorhiza ends the sensu stricto germination in the Poaceae. So far, experiments with caryopses have relied on different germination criteria: coleorhiza emergence (CE) through the coat (Gubler et al. 2008), radicle emergence (RE) through the coleorhiza (Gendreau et al. 2008), or both (Jacobsen et al. 2013; González-Calle et al. 2015).

Caryopses of the wild oat (*Avena fatua*), a highly pernicious annual weed which infests major cereals worldwide leading to huge losses (Simpson 2007), show physiological dormancy expressed as inability to complete germination at temperatures exceeding 12 °C (Adkins and Peters 2001) or 20 °C (Kępczyński et al. 2013). Thus, dormancy is a relative phenomenon. Primarily, dormant *A.* *fatua* florets/caryopses accumulate in soil to form a soil bank in which they remain viable for several years, thereby posing a considerable challenge to the control of the weed (Simpson 2007). The caryopsis dormancy can be released during dry storage (after-ripening) at 20–40 °C (Foley 1994; Kępczyński and Van Staden 2012). *Avena* *fatua* dormancy has also been shown to be released by gibberellin GA_3_ (Adkins et al. 1986; Merritt et al. 2006; Kępczyński et al. 2006, 2013), plant-derived smoke (Adkins and Peters 2001; Kępczyński et al. 2006, 2010), karrikin 1 (KAR_1_) presented in smoke (Daws et al. 2007; Stevens et al. 2007; Kępczyński et al. 2010, 2013), and hydrogen peroxide (Cembrowska-Lech et al. 2015)*.* Although exogenous ethylene was unable to completely remove dormancy in caryopses, endogenous ethylene was found to be necessary to break dormancy by KAR_1_ (Kępczyński and Van Staden 2012).

It was also postulated that endogenous gibberellins are required to release dormancy by KAR_1_ in *A. fatua* caryopses (Kępczyński et al. 2013). The stimulatory effect of KAR_1_ and GA_3_ on the completion of germination in dormant caryopses is associated with the non-transcriptional and transcriptional activation of ACC synthase (ACS) and ACC oxidase (ACO) enzymes, respectively, and with modulation of the ethylene sensitivity through control synthesis of ethylene receptors (Ruduś et al. 2019). *Avena* *fatua* caryopses are used in our research as a model system in which to study the mechanism of dormancy release (Kępczyński 2018), since the knowledge obtained might prove useful for developing a strategy for combatting this troublesome weed.

The role of ABA in regulating seed dormancy has been widely studied, mainly in the dicotyledonous seeds (Rodríguez-Gacio et al. 2009; Arc et al. 2013). Less information is available on monocot seeds, especially weeds*.* There are no data on the interaction between KAR_1_ and ABA in seeds, except for those of *Arabidopsis* (Nelson et al. 2009). The knowledge on a relationship between KAR_1_ and ABA in regulating germination of dormant *A.* *fatua* caryopses is far from satisfactory as well. Moreover, the knowledge of the cell cycle in relation to primary dormancy release in seeds is very poor. There is only modest information on the role of the cell cycle in dormancy of tomato seeds (De Castro et al. 2001) and barley grains (Gendreau et al. 2012). Our earlier papers (Cembrowska-Lech and Kępczyński 2016, 2017) reported that both smoke and KAR_1_ applied to dormant *A.* *fatua* caryopses activated the cell cycle before RE. However, data on the regulation of the *A.* *fatua* caryopsis cell cycle by ABA are completely missing.

The aim of this work was to examine the relationship between KAR_1_ or dry after-ripening and ABA in the regulation of *A.* *fatua* caryopsis germination. To this end, the present study was conceived to: (1) determine effects of KAR_1_ and ABA on two stages of germination, coleorhiza emergence (CE) and radicle emergence (RE), of dormant and dry after-ripened caryopses, respectively; (2) investigate the ABA content changes in embryos during germination of dormant caryopses in the presence of KAR_1_; (3) compare the ABA contents in the coleorhiza and radicles of embryos from untreated and KAR_1_-treated dormant caryopses; (4) determine in the ABA content in embryos from after-ripened dry caryopses and from after-ripened caryopses at an early stage of germination; (5) find out whether (a) germination of dry after-ripened caryopses is associated with activation of the cell cycle, and (b) ABA influences the cell-cycle progression.

The results are expected to provide new insights into the role of ABA in dormancy release (in relation to KAR_1_ and after-ripening) and germination of *A.* *fatua* caryopses used as a model in which to investigate the dormancy mechanism in agricultural weed seeds, with a special reference to karrikin.

## Material and methods

### Plant material

*Avena fatua* L. (wild oat) spikelets were collected on between July 20, 2015 and July 18, 2017, during the time of their natural dispersal, in the vicinity of Szczecin (53.4285°N, 14.5528°E) (Poland). The spikelets containing florets were dried at room temperature for 7 days to constant moisture (ca. 11%), and were stored at − 20 °C until required. Partly and fully after-ripened (non-dormant) caryopses were collected to store dry dormant florets in open air under ambient relative humidity in darkness at 35 °C for up to 8 and 16 weeks, respectively. All the experiments and measurements, except for flow cytometry, involved caryopses from florets collected in 2015.

### ***Caryopsis germination and karrikin 1 (KAR***_***1***_***) or ABA treatments***

A total of 25 caryopses (florets without the lemma and palea), in 3 replicates each, were incubated in the dark at 20 °C in Petri dishes (ø 60 mm) on a single layer of filter paper (Whatman No. 1) moistened with 1.5 mL distilled water or a solution of KAR_1_ or ABA. Caryopses showing CE from the coat and RE through the coleorhiza were counted every day until day 5 of germination, or else CE or RE were determined only after 5 days. The handling was performed under a safe (not affecting germination) green light at 0.5 µmol m^−2^ s^1^.

In the experiment with karrikin applied after 0 and 16 weeks of floret dry storage, the caryopses were incubated on water and KAR_1_ solutions (10^–9^, 3 × 10^–9^, 10^–8^ M) for 5 days. KAR_1_ used in the treatments was synthesized according to Nagase et al. (2008). The caryopses from florets after-ripened for 8 or 16 weeks were incubated on water and ABA solutions (10^–5^, 10^–4^ M) for 5 days.

In another experiment, the caryopses from florets dry after-ripened for 16 weeks (non-dormant) were pre-incubated on water for 6 h and then incubated on water and ABA solutions (10^–5^, 10^–4^ M) for 5 days.

### ABA content measurement

Dormant caryopses (25 in each of the 3 replicates) were incubated on water or 3 × 10^–9^ M KAR_1_ solution for 0, 6, 18, 24, 30, and 36 h. The caryopses, dry after-ripened for 16 weeks (25 in each of the 3 replicates), were incubated on water for 18 h. Embryos (the coleorhiza and radicles in one experiment) were isolated before or after the period of incubation. The material was lyophilized, and 10 mg samples were pulverized with zirconia beads (MM400, Retch, Germany). ABA was analyzed as described by Dziurka et al. (2019), with some modifications. Samples were spiked with 20 pmol of heavy-labelled internal standard ([^2^H_6_] ABA) and triple extracted in the extraction buffer (methanol/water/formic acid, 15/4/1, by vol.). The combined supernatants were evaporated under N_2_, resuspended in 3% methanol in 1 M formic acid, and cleaned on hybrid SPE cartridges (BondElut Plexa PCX, Agilent USA). The analyses were run in the MRM mode of an UHPLC apparatus (Agilent Infinity 1260, Agilent, Germany) coupled with a triple quadruple MS/MS mass spectrometer (6410 Triple Quad LC/MS, Agilent, USA) equipped with an electrospray ionization (ESI) source. The separation was achieved on an Ascentis Express RP-Amide analytical column (2.7 μm, 2.1 mm × 75 mm; Supelco). Quantification was based on calibration curves obtained for pure ABA standards, taking into account the recovery of the internal standard ([^2^H_6_] ABA). The hormone standards were obtained from Olchemim (Olomouc, Czech Republic), while other chemicals were purchased from Sigma-Aldrich (Poznań, Poland).

### **Nuclear DNA determination**

The nuclear DNA contents in radicle tips with coleorhiza (RC) were determined using flow cytometry. For the cell-cycle activity determination, dormant caryopses (25 in each of the 5 replicates) were incubated at 20 °C for 20, 24, 36, and 48 h in the dark on distilled water. Caryopses after-ripened for 16 weeks were germinated on water or in the presence of ABA (10^−4^ M) under identical conditions for 20, 24, 30, 36, and 48 h. Twenty-five RCs were isolated from the germinated caryopses and, using a razor blade, were chopped and placed in 2 mL nucleus isolation buffer (45 mM MgCl_2_, 30 mM sodium citrate, 20 mM MOPS, 0.1% Triton X-100, and 2 µg/mL DAPI) (Galbraith et al. 1983) for 10 min at 25 °C. Subsequently, the suspension was passed through a 20 µm nylon mesh. The DAPI-stained nuclei were analyzed using a Partec PAII flow cytometer (Partec). The populations of 2C and 4C nuclei were counted in 10,000 nuclei. Changes in the number of nuclei observed were related to the radicles, as it has been shown that the barley coleorhiza cells do not undergo division (Barrero et al. 2009).

### **Statistical treatment**

The means were tested for significance of differences using one- or two-way analysis of variance, ANOVA (Statistica for Windows v. 10.0, Stat-Soft Inc., Tulsa, OK, USA). Duncan’s multiple range test was used to identify the significantly different (*P* ≤ 0.05) mean values.

## Results

### ***Effects of KAR***_***1***_*** on coleorhiza and radicle emergence in dormant and after-ripened caryopses***

**Fig. 1:**
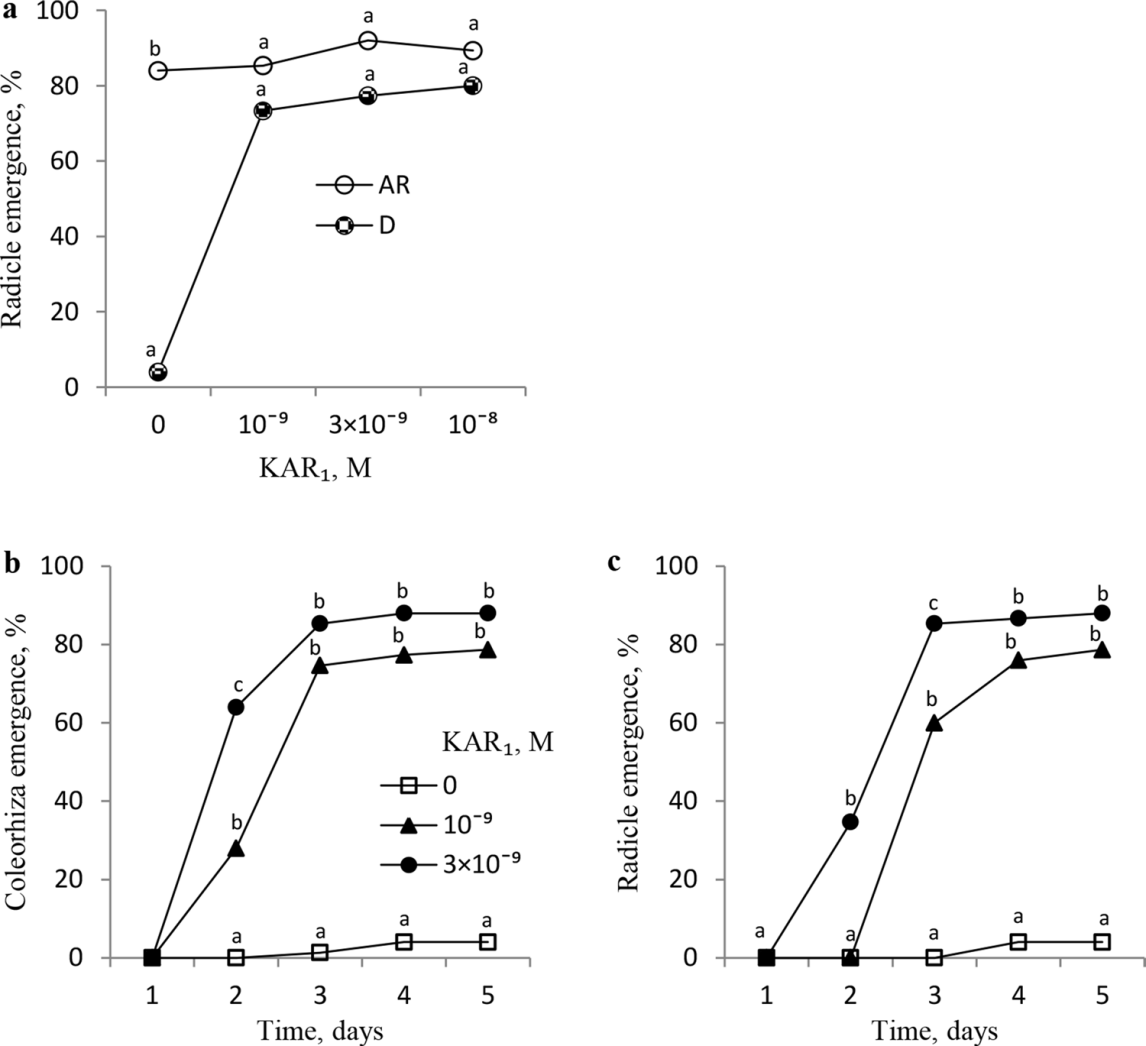
Effects of KAR_1_ on coleorhiza (**b**) and radicle emergence (**a, c**) in *A.* *fatua* caryopses dormant (**a, b, c**) and after-ripened for 16 weeks (**a**). **a** D, dormant, AR, after-ripened; radicle emergence was determined after 5 days. One-way ANOVA with Duncan’s post hoc test was used to test for significance of differences. Means denoted by different letters (a-e) are significantly different (*P* < 0.05; *n* = 3)

Effects of KAR_1_ on RE in dormant and dry after-ripened (non-dormant) caryopses for 16 weeks at 35 °C were compared after 5 days of germination. Dormant caryopses and almost all the after-ripened ones were unable and able (ca. 80%) to emerge radicles, respectively (Fig. 1a). KAR_1_ stimulated RE in the dormant caryopses; the extent of the influence was similar regardless of the concentration used, with ca. 80% of caryopses completing germination. Effects of KAR_1_ on the CE and RE after various germination times were also determined. During the entire incubation of dormant caryopses on water, the coleorhiza or radicle emergence was observed in as few as ca. 5% of the caryopses (Fig. 1 b, c). After 2 days, KAR_1_ at a concentration of 10^–9^ M increased the coleorhiza emergence, and was more effective at a higher concentration, 3 × 10^–9^ M (Fig. 1b). The extension of germination time in the presence of KAR_1_ at both concentrations resulted in an almost complete CE. KAR_1_ also increased the radicle emergence through the coleorhiza (Fig. 1c); RE was not affected after 2 days at 10^–9^ M, but at 3 × 10^–9^ M, radicles were observed to have emerged. After 3 days, the difference in response depending on the concentration used was observed; at 10^–9^ and 3 × 10^–9^ M, a distinct and complete RE was observed, respectively.

### ***ABA contents in embryos, coleorhiza, and radicles during germination of dormant caryopses in the absence or in the presence of KAR***_***1***_

**Fig. 2:**
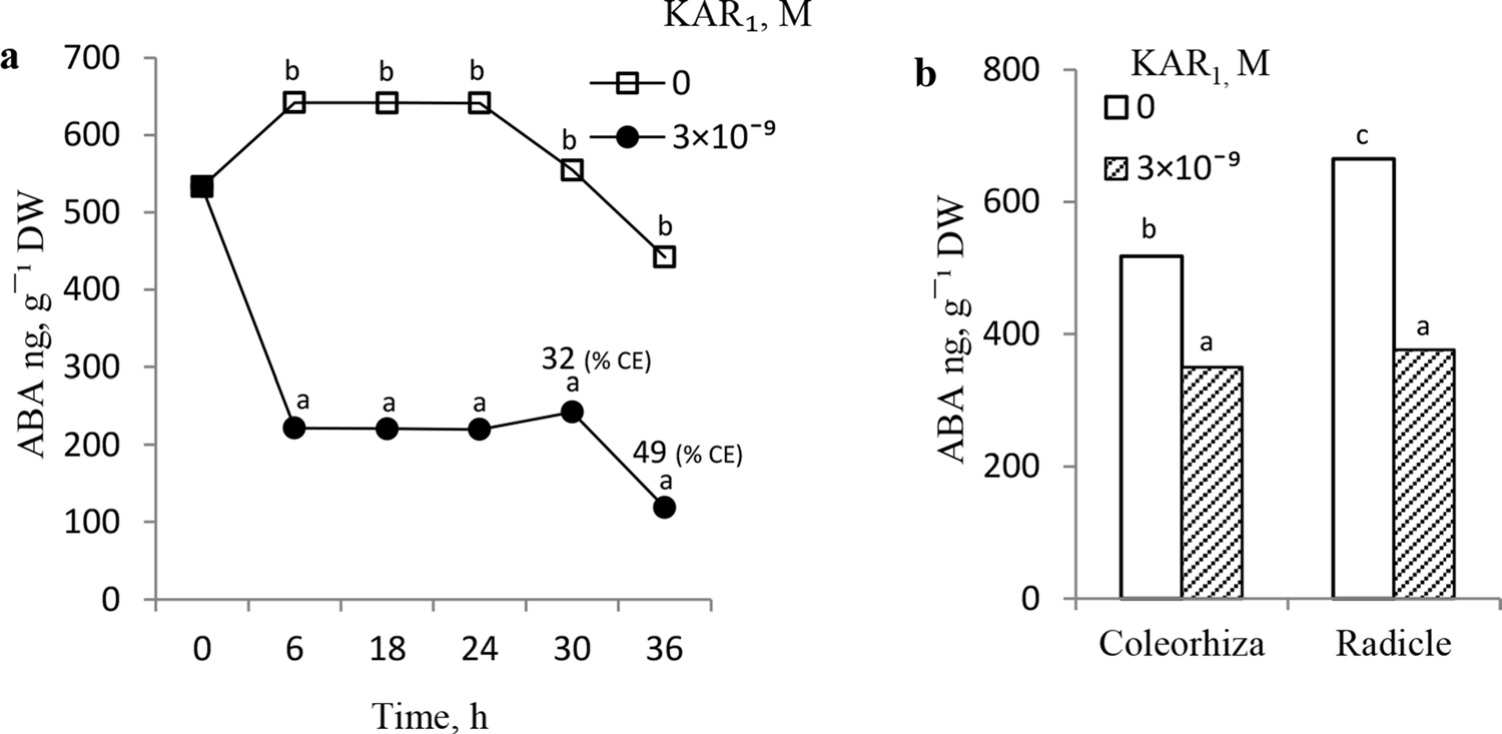
Effects of KAR_1_ on ABA content in embryos isolated from dormant *A. fatua* caryopses germinated for various times (**a**) and in coleorhiza or radicles of caryopses germinated for 24 h (**b**). CE, coleorhiza emergence. One-way (**a**) or two-way (**b**) ANOVA with Duncan’s post hoc test was used to test for significance of differences. Means denoted by different letters (a–e) are significantly different (*P* < 0.05; *n* = 3)

The ABA content was determined in embryos isolated from dormant caryopses kept to germinate on water or in 3 × 10^–9^ M KAR_1_ for various periods up to 36 h. During germination of the dormant caryopses on water, during the first 6 h, the embryos’ ABA content increased and then remained constant for up to 24 h (Fig. 2a). Extension of the germination time to 36 h resulted in a progressive reduction of the ABA content. The presence of KAR_1_ during the initial 6 h of germination sharply reduced the ABA level. Subsequently, the ABA content was constant up to 30 h, to drop thereafter, as the germination time was extended by an additional 6 h. The dormant caryopses were not able to complete germination on water for up to 36 h: neither the coleorhiza nor the radicle appeared. Germination of caryopses in the presence of KAR_1_ for 30 or 36 h resulted in approximately 30 or 50% CE, respectively (Fig. 2a). The effect of KAR_1_ applied during a 24 h-long germination of dormant caryopses on the ABA content in the coleorhiza and radicles was also analyzed. The ABA content in radicles of the embryos isolated from caryopses kept to germinate on water was a little higher than that in the coleorhiza (Fig. 2b). Germination of caryopses in the presence of KAR_1_ decreased the ABA content in the coleorhiza and radicles.

### **Effects of ABAon coleorhizaand radicleemergence inafter-ripenedcaryopses**

**Fig. 3:**
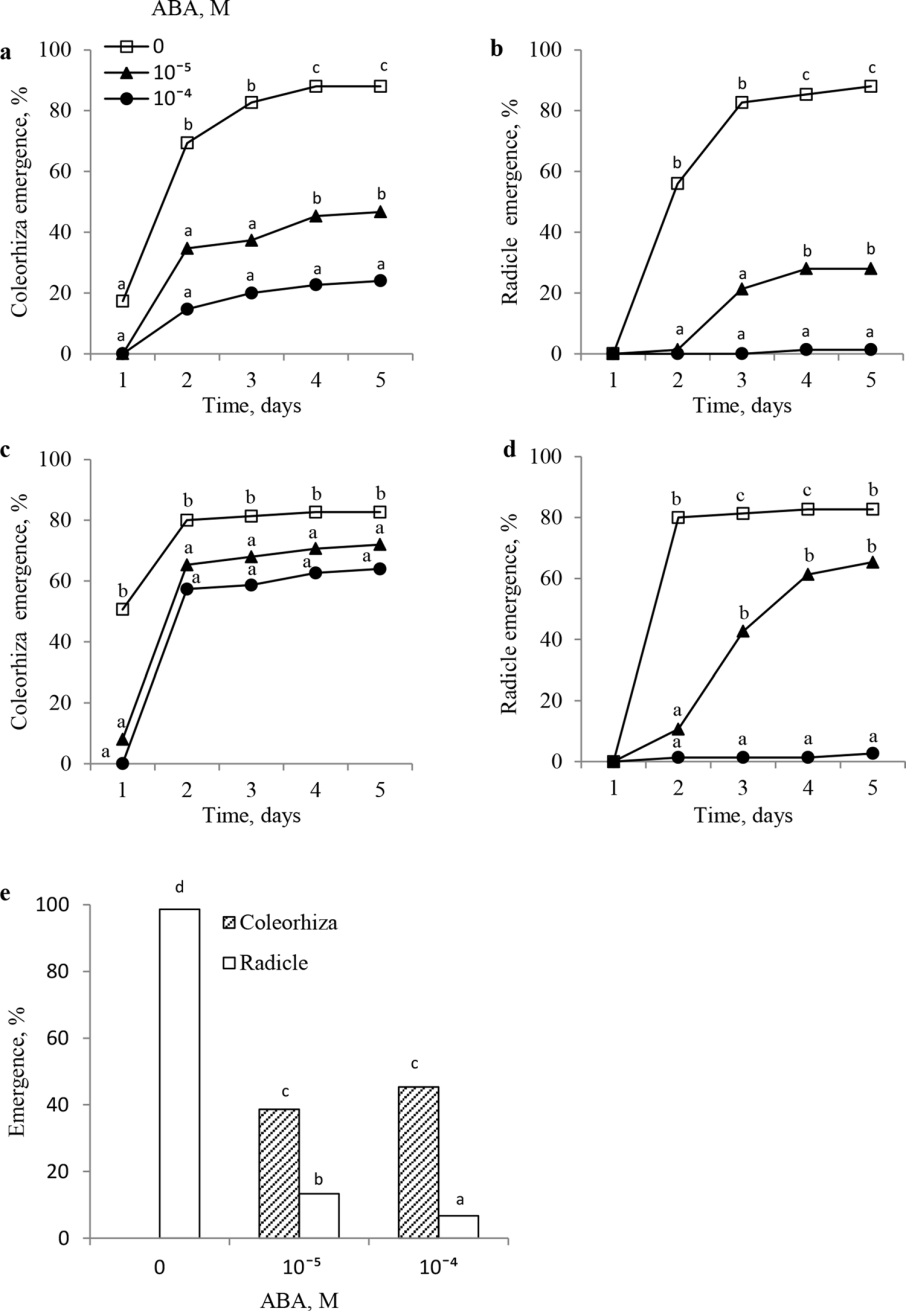
Effects of ABA on coleorhiza (**a**, **c**, **e**) and radicle (**b**, **d**, **e**) emergence in *A.* *fatua* caryopses after-ripened for 8 (**a**, **b**) and 16 (**c**, **d**, **e**) weeks. **e** After-ripened caryopses were pre-incubated on water for 6 h and then germinated on water or ABA solutions for 5 days. One-way (**a**, **b**, **c**, **d**) or two-way (**e**) ANOVA with Duncan’s post hoc test was used to test for significance of differences. Means denoted by different letters (a–e) are significantly different (*P* < 0.05; *n* = 3)

Both CE and RE were determined after germination on water or on ABA solutions in caryopses after-ripened for 8 and 16 weeks. The caryopses after-ripened for 8 weeks and kept to germinate on water for 1 or 2 days showed CE of ca. 20 and 70%, respectively (Fig. 3a). Extension of the germination time for up to 4 days resulted in an almost complete CE. At both concentrations, ABA was found to inhibit CE markedly during the entire period of germination. After a 5d germination on water, RE was similar to that of CE (almost complete), while ABA applied at concentrations of 10^–5^ and 10^–4^ M, markedly or completely inhibited RE (Fig. 3b). Germination of, after-ripened for 16 weeks caryopses, on water for 1d resulted in ca. 50% (Fig. 3c). Extension of the germination time to 2 days resulted in a complete CE. At both concentrations (10^–5^ and 10^–4^ M), ABA completely inhibited CE after 1 day. From day 2, CE was lower than that of the caryopses germinated in water. Germination for 2d on water resulted in an almost complete RE (Fig. 3d). ABA applied at a concentration of 10^–5^ M was found to inhibit RE almost completely for up to 2 days. A weaker inhibitory effect was still evident for up to 4 days. At the ABA concentration of 10^–4^ M, the radicles failed to protrude through the coleorhiza even after a 5d-long germination. Effects of ABA on CE and RE of caryopses after-ripened for 16 weeks and pre-incubated for 6 h on water were also determined. After 5 days of germination on water, CE and RE were complete (Fig. 3e). The ABA concentrations of 10^–5^ and 10^–4^ M resulted in a marked CE reduction. Again, a much stronger inhibitory effect of ABA was seen in RE. A stronger inhibitory effect of RE was visible in the presence of a higher ABA concentration.

### ABA contents in embryos from after-ripened dry caryopses and after-ripened caryopses germinated on water

**Fig. 4:**
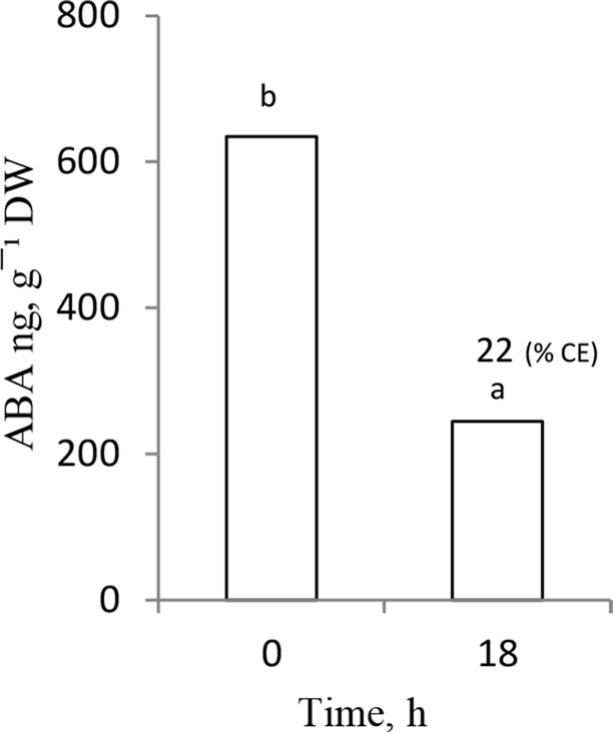
ABA contents in embryos from dry and 18 h-germinated *A.* *fatua* caryopses after-ripened for 16 weeks. One-way ANOVA with Duncan’s post hoc test was used to test for significance of differences. Means denoted by different letters (a–e) are significantly different (*P* < 0.05; *n* = 3)

The ABA content was determined in embryos isolated from dry caryopses and in those kept, for 18 h, to germinate on water; all the caryopses had been earlier after-ripened for 16 weeks. The embryos’ ABA content strongly declined when after-ripened caryopses were germinated on water for 18 h (Fig. 4). The ABA content in embryos from water-germinated caryopses was ca. 3 times lower than in embryos of the dry caryopses. In this experiment, ca. 20% of the caryopses with CE were observed after germination on water.

### Nuclear DNA contents in radicle tips with coleorhiza (RC) after germination of dormant caryopses on water or in after-ripened caryopses germinated in the absence or in the presence of ABA

**Fig. 5:**
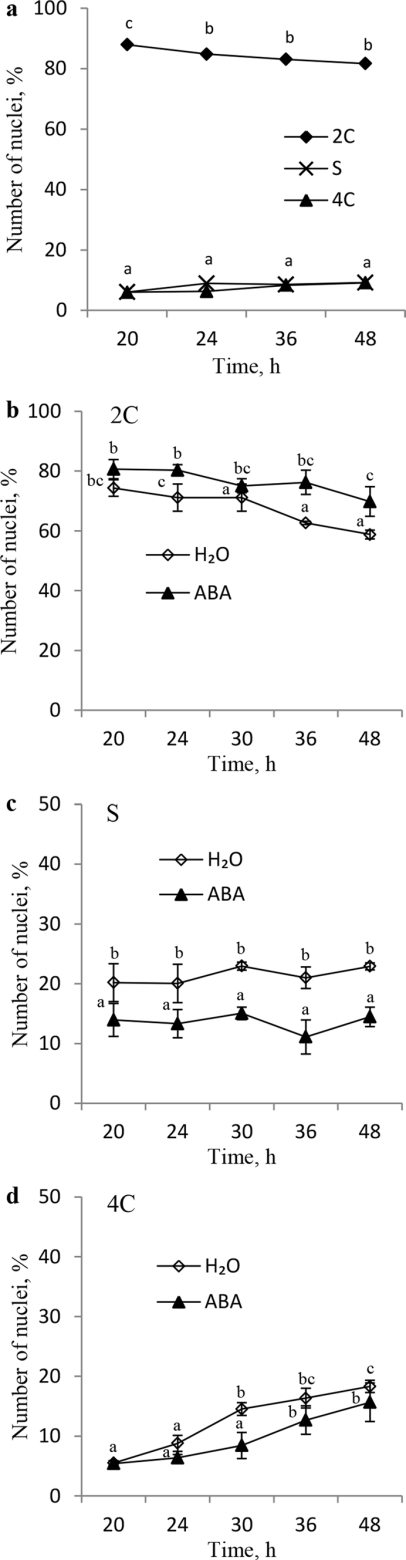
Nuclear DNA content in radicle tips with coleorhiza after germination of dormant *A.* *fatua* caryopses on water (**a**) and after-ripened caryopses for 16 weeks (**b**, **c**, **d**) germinated in the absence or in the presence of ABA (10^–4^ M) for various times. Coleorhiza emergence after 36 and 48 h germination of after-ripened caryopses on water was 42 and 40%, respectively. Radicle emergence after 48 h germination on water was 18%. Coleorhiza emergence after 36 and 48 h germination of after-ripened caryopses in the presence of ABA was 8 and 24%, respectively. Two-way ANOVA with Duncan’s post hoc test was used to test for significance of differences. Means denoted by different letters (a–e) are significantly different (*P* < 0.05; *n* = 5)

The nuclear DNA content was determined in RC of dormant caryopses incubated on water for various periods as well as in dry caryopses after-ripened for 16 weeks (non-dormant) and germinated in the absence or in the presence of ABA. Germination of dormant caryopses kept on water for 20 h was associated with 88% of nuclei showing the 2C signal (Fig. 5a). Extension of the germination time to 48 h decreased the signal down to 82%. The percentage of nuclei at S and 4C was much lower (6.1–9.1%) during the 48 h germination. When after-ripened caryopses were germinated on water for the same length of time, progressive changes in the percentage of nuclei at 2C and 4C were observed (Fig. 5b, d). The 2C signal was weaker in RC from the after-ripened caryopses than from the dormant ones, and decreased from ca.74 to 59% during 48 h-long germination (Fig. 5b). The percentage of 4C nuclei increased from ca. 5 to 18 (Fig. 5d), and was slightly higher than in the dormant caryopses germinated for the same length of time (Fig. 5a). The S signal in RC from the after-ripened caryopses was ca. 20% during the whole period of germination (Fig. 5c), but was stronger than that in the dormant caryopses (Fig. 5a). Germination of non-dormant caryopses in the presence of ABA resulted in a decrease in the 2C and an increase in the 4C nuclei populations during 48 h. The percentages of nuclei at Sand 4C in contrast to 2C were lower during the whole period of germination when an ABA solution was used instead of water. After 36 h of germination of non-dormant caryopses in the absence or in the presence of ABA, CE was observed to amount to 42 and 8%, respectively. Extension of the germination time for up to 48 h increased CE to 24% in the ABA-treated caryopses and resulted in 18% RE in the non-treated ones.

## **Discussion**

### ***Responses of dormant and after-ripened caryopses to KAR***_***1***_*** and ABA***

Earlier studies (Kępczyński et al. 2010, 2013; Kępczyński and Van Staden 2012) demonstrated that freshly harvested *A.* *fatua* caryopses were hardly able to complete germination at 20 °C. Therefore, these caryopses are considered to be primarily dormant. This primary dormancy can be removed by various agents, including dry after-ripening and KAR_1_, the treatments making it possible for all or almost all the caryopses to complete germination (Kępczyński 2018; Fig. 1). Cereal caryopsis germination has been often assessed, including in some of our earlier studies on *A.* *fatua* (Kępczyński and Van Staden 2012; Kępczyński et al. 2013), based on the radicle emergence (a germination criterion) which ends the *sensu stricto* germination, like in the dicot seeds (Bewley 1997). The completion of germination in grasses, e.g., *Brachypodium distachyon* (González-Calle et al. 2015), was described as a two-stage process with the CE as the first stage, followed by the RE through the coleorhiza, the second one. The present study employed both CE and RE to assess germination. KAR_1_ turned out to stimulate not only *A.* *fatua* caryopsis RE, but also CE, the earlier stage of germination (Fig. 1). Effects of KAR_1_ on CE were stronger than on RE, indicating that the coleorhiza is more sensitive to the compound than the radicle. Since coleorhiza growth in several cereals is associated with cell elongation (Sargent and Osborne 1980; Barrero et al. 2012), the KAR_1_ effect on CE probably involves stimulation of cell elongation. The coleorhiza is recognized as a structure which protects the emerging radicle (Sargent and Osborne 1980). It is also considered to play a major role in causing dormancy by acting as a barrier to RE (Barrero et al. 2009). In light of the above, it could be presumed that the grass caryopsis dormancy release by various factors, e.g., by KAR_1_ in *A.* *fatua*, involves the induction of the coleorhiza cell elongation and separation.

Abscisic acid is known to control dormancy in caryopses of several cereals. The inability of caryopses to complete germination is associated with the ABA level being too high (Fidler et al. 2015; Rodríguez et al. 2015). Consequently, the dormant *A.* *fatua* caryopses were not able to complete germination (Fig. 1) because of the high ABA level during germination (Fig. 2a). KAR_1_ strongly reduced the ABA content in embryos during the whole period of germination, before RE. In contrast, the ABA level prior to RE in *Arabidopsis* seeds was unaffected by KAR_1_ (Nelson et al. 2009). As early as after a 6 h-long germination, the ABA content was suddenly reduced, by a factor of about 3, under the influence of KAR_1_ (Fig. 2a). The inhibitor contents in embryos from untreated and KAR-treated caryopses differed by a factor of 3 after 18 and 24 h of germination. After 36 h, when CE of the KAR-treated caryopses was ca. 50%, the difference increased to being fourfold. It seems undeniable that the ABA content reduction due to the presence of KAR_1_ facilitated CE and also RE, and hence termination of phase II, thus the dormancy release. The ABA contents in both the coleorhiza and the radicle of dormant caryopses germinated for 24 h on water were high (Fig. 2b). The extent of KAR_1_ impact on the ABA content depended on the part of the caryopsis. KAR_1_ reduced the ABA content both in the coleorhiza and in the radicle, but more so in the latter, which may suggest a faster ABA catabolism in the radicle. It has been previously demonstrated that less ABA was contained in both the coleorhiza and the radicle in after-ripened, non-dormant, barley grains than in the dormant ones germinated for 48 h (Barrero et al. 2009). In the barley caryopsis coleorhiza, after-ripening promoted ABA catabolism by controlling the gene-encoding ABA hydroxylase (HvABA8, OH-1). Barrero et al. (2009) considered ABA as the key factor responsible for the lack of the CE and as playing the main role in the maintenance of dormancy by preventing the RE. Thus, in both barley and *A.* *fatua*, a reduction of the ABA level in the coleorhiza seems to be very important in dormancy release probably by leading to the coleorhiza degradation and, consequently, to RE. Studies with *Brachypodium distachyon* showed that the weakening of the coleorhiza cell walls by endo-β-mannanases facilitated the RE (González-Calle et al. 2015). Given that KAR_1_ affects the ABA content not only in the coleorhiza, but also in the radicle, it has to be contended that the RE requires not only an ABA-level reduction in the coleorhiza, which presumably enables the loosening of the coleorhiza cell walls, but also in the radicle. The possibility that the RE may depend not only on the softening of the coleorhiza but also on the expansive force of the radicle has been previously suggested for barley (Barrero et al. 2009).

Dormancy in *A. fatua* caryopses can be completely removed not only by KAR_1_ but also by a 16-week-long dry after-ripening. The effect was observed when these caryopses were kept to germinate on water; both CE first and the complete RE later were then possible (Fig. 3). The after-ripened, thus non-dormant, caryopses were capable of completing germination as a result of a reduction in their ABA level; the ABA content in embryos after 18 h germination of the after-ripened caryopses (Fig. 4) was 3 times lower than that in embryos from the dormant caryopses after an identical period of germination on water (Fig. 2a). Thus, similarly to sunflower (Xia et al. 2019) and barley (Jacobsen et al. 2002; Millar et al. 2006), after-ripening of *A.* *fatua* caryopses results in a reduction of the ABA content during the early stage of germination. Moreover, the ABA content in embryos from the after-ripened caryopses germinated for 18 h on water was similar to that in dormant caryopses germinated in the presence of KAR_1_ for the same length of time. The data indicate that, regardless of the dormancy-breaking factor used (after-ripening or KAR_1_), the effect was associated with a reduction in the ABA content during the early stage of germination. The reduction in the ABA level can be related to the stimulation of ABA conversion to phaseic acid, as shown earlier (Cembrowska-Lech and Kępczyński 2016). A progressive release of dormancy over time of after-ripening was correlated with a decrease in the coleorhiza response to ABA (Fig. 3a, c), indicating that dormancy removal is associated also with a reduction in the sensitivity to ABA. A decreased coleorhiza sensitivity to ABA due to after-ripening has been reported also for barley grains (Barrero et al. 2009). Likewise, the radicle sensitivity to ABA decreased when after-ripening was prolonged (Fig. 3b, d). Moreover, the radicle turned out to be more sensitive to ABA than the coleorhiza, regardless of the after-ripening duration (Fig. 3). The experiment with treating the non-dormant caryopses, pre-incubated on water, with ABA also showed a higher sensitivity to the inhibitor of the radicle, compared to the coleorhiza (Fig. 3c). Therefore, the higher sensitivity to ABA of the radicle than the coleorhiza is perhaps related to a little higher ABA level in the former, compared to that in the latter (Fig. 2b). Data on the *A.* *fatua* caryopses may, similarly to the barley experiment (Barrero et al. 2009), indicate the importance of reduced coleorhiza sensitivity to ABA as the mechanism of dormancy release by after-ripening. In sunflower seeds, after-ripening is mediated by a decline in both the ABA content and sensitivity, which is related to relocation and degradation of the effector, the Absciscic Acid-Insensitive 5 protein (Xia et al. 2019).

### Cell cycle activity in relation to dormant and after-ripened untreated or ABA-treated caryopses

Like in earlier experiments (Cembrowska-Lech and Kępczyński 2016, 2017), the percentage of nuclei at phases G_1_, S, and G_2_ did not change during germination of dormant caryopses, incapable of RE (Fig. 5a). It had been also found earlier that the DNA synthesis did not increase during germination of dormant *A.* *fatua* embryos (Elder and Osborne 1993). The data obtained confirm that dormancy in caryopses of the species is associated with inhibition of the cell-cycle activity. Similarly to the *A.* *fatua* caryopses, dormant germinating tomato seed cells were arrested at phase G_1_ (De Castro et al. 2001). However, the cell cycle was partially reactivated in embryos during germination of dormant barley grains (Gendreau et al. 2012). KAR_1_, GA_3_, and smoke water, germination stimulants in dormant *A.* *fatua* caryopses (Kępczyński 2018), were found to reduce the percentage of nuclei at G_1_ and to increase the proportion of those at S and G_2_ during germination (Cembrowska-Lech and Kępczyński 2016, 2017). After-ripening, which allows dormancy release and hence germination completion by reducing the ABA level, was observed to decrease the number of cells at phase G_1_ and to increase the number of cells at S and G_2_ (Fig. 5b–d). It can be then concluded that dormancy release by after-ripening involves inducement of an ability to decrease the ABA content during germination, which enables the cell-cycle activation. Activation of the cell cycle in non-dormant germinating barley was related to a decrease in the ABA content (Gendreau et al. 2008). The cell-cycle activation by benzyladenine in maize seeds was associated with stimulation of germination (Sanchez et al. 2005). As stated previously, a reduction of the ABA content prior to the RE in the KAR_1_-treated *A.* *fatua* dormant caryopses was associated with the cell-cycle activation (Cembrowska-Lech and Kępczyński 2016; Fig. 2a). Thus, regardless of the dormancy-breaking agent (KAR_1_, after-ripening), the ABA content was found to decrease, which results in activating the cell cycle before germination is completed. ABA inhibited germination of the non-dormant *A.* *fatua* caryopses (Fig. 3) and decreased the number of nuclei at S and at G_2_ (Fig. 5c, d). Thus, the inhibitory effect of ABA on germination is at least in part based on the cell-cycle activity regulation. Likewise, ABA-induced inhibition of seed germination in maize and barley was associated with a delay in the cell-cycle activation (Sánchez et al. 2005; Gendreau et al. 2008). Germination inhibition by ABA is probably associated with additional effects, since, despite the presence of the hormone, some cell-cycle activation was found to occur. Earlier experiments with dormant and ABA-treated non-dormant barley grains showed that the inability to complete germination and germination inhibition, respectively, resulted in blocking of the G_2_/M and G_1_/S transition (Gendreau et al. 2012). The inhibitory effect of ABA did not emulate the inhibition of germination associated with dormancy at the level of gene expression. Therefore, it was also suggested that regulation of the cell cycle during germination of dormant barley grains is only in part controlled by the ABA metabolism (Gendreau et al. 2012).

In conclusion, the *A*.* fatua c*aryopsis dormancy release by KAR_1_ is associated with a reduction of the ABA content in embryos, coleorhiza, and radicles prior to the CE. The dormancy release by after-ripening is related to the coleorhiza being more sensitive to KAR_1_ and less sensitive to ABA than the radicles, and to a decrease in the embryo ABA content prior to the RE. The inability to complete germination because of dormancy or the presence of ABA during germination in after-ripened caryopses is related to total or partial inhibition of the cell-cycle activation, respectively.

#### *Author contribution statement*

JK conceived and designed the research, interpreted results, and wrote the manuscript. AW conducted physiological experiments. MD carried out GC analysis. All authors read, reviewed, and approved the manuscript.

## Abbreviations

KAR_1_: Karrikin 1
CE: Coleorhiza emergence
RE: Radicle emergence
